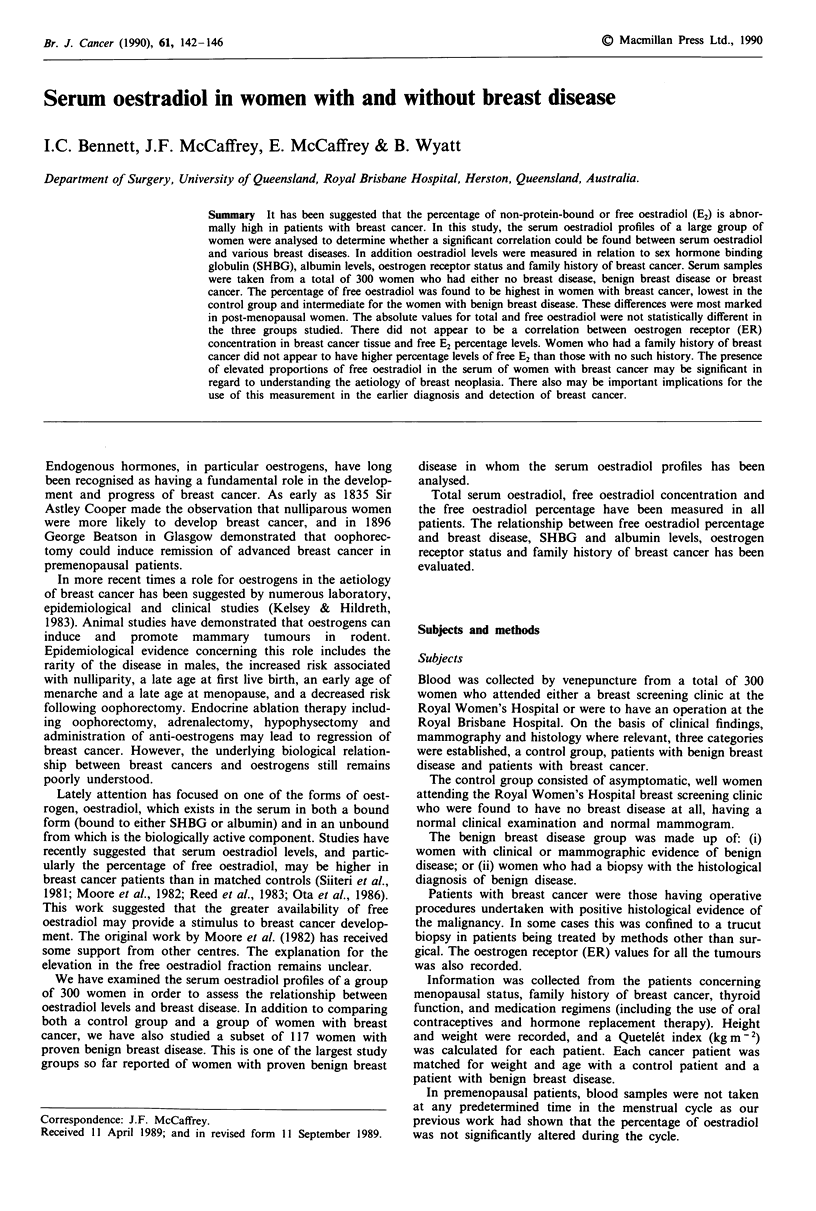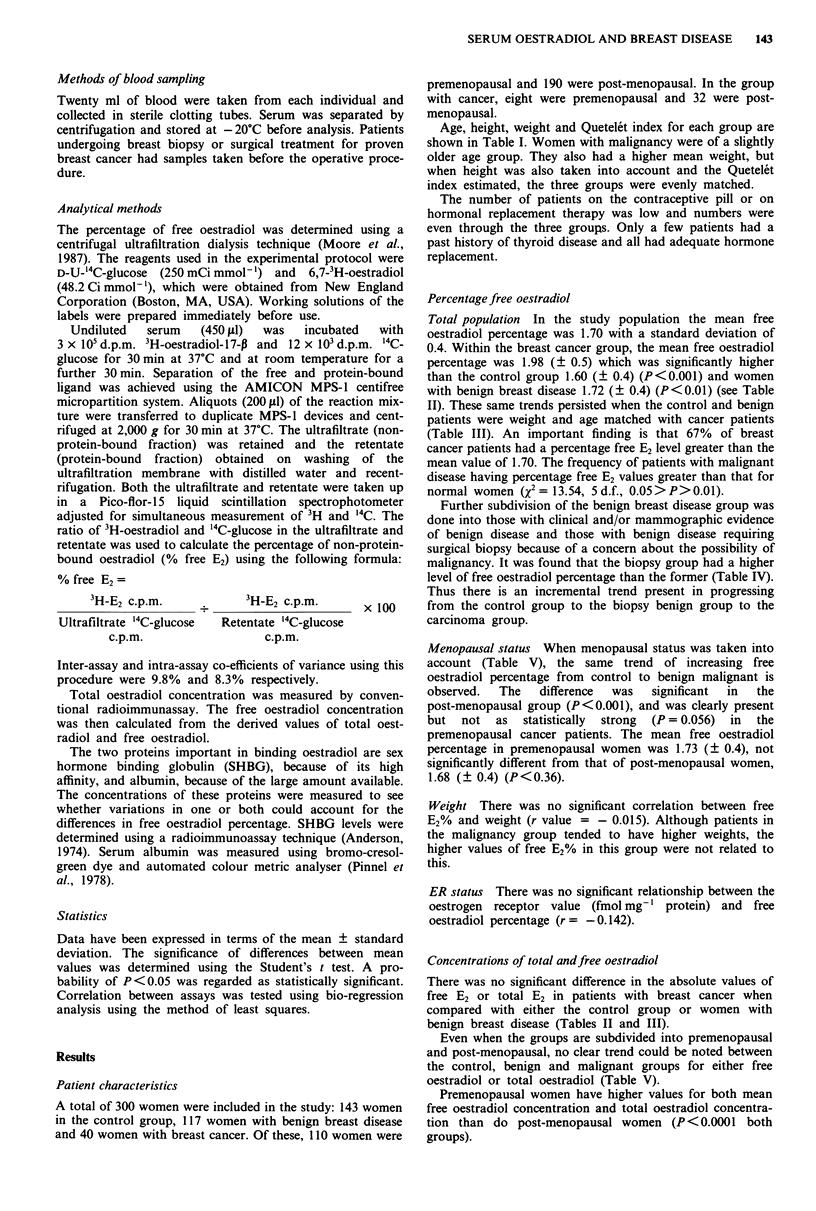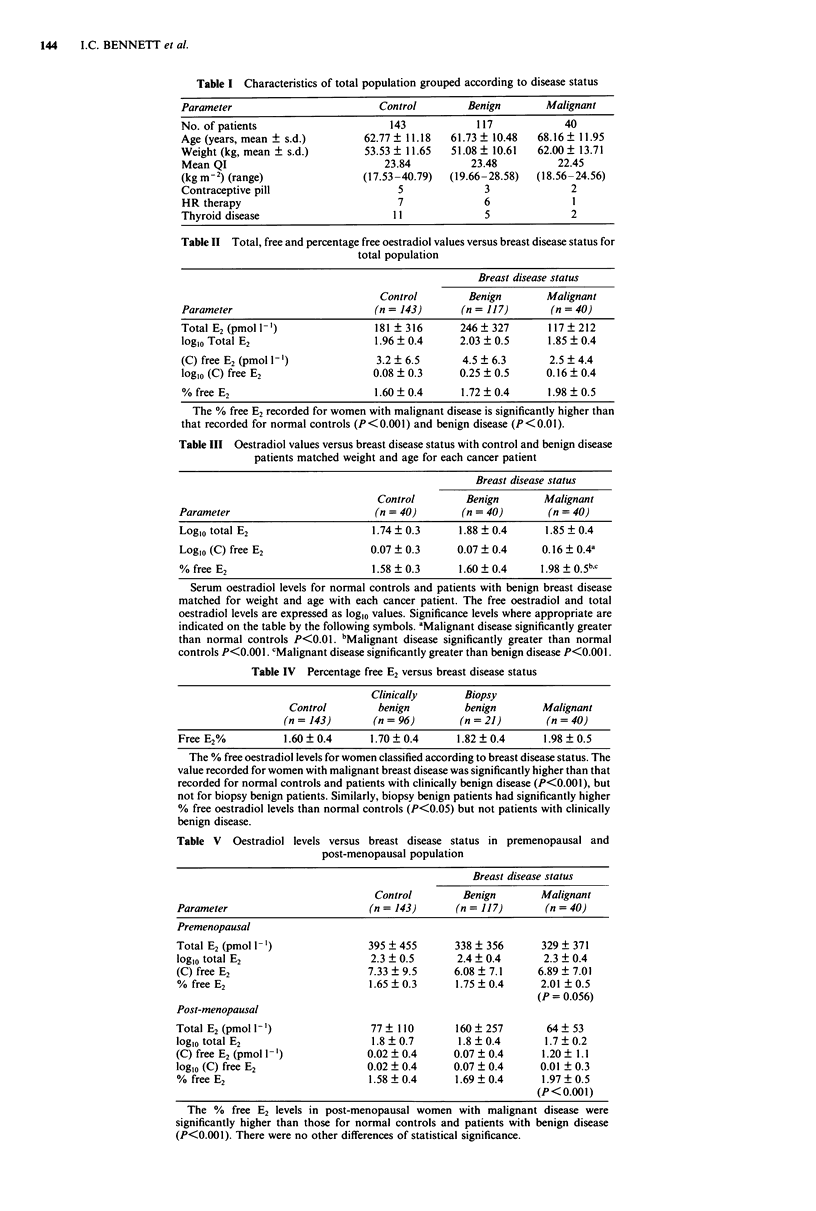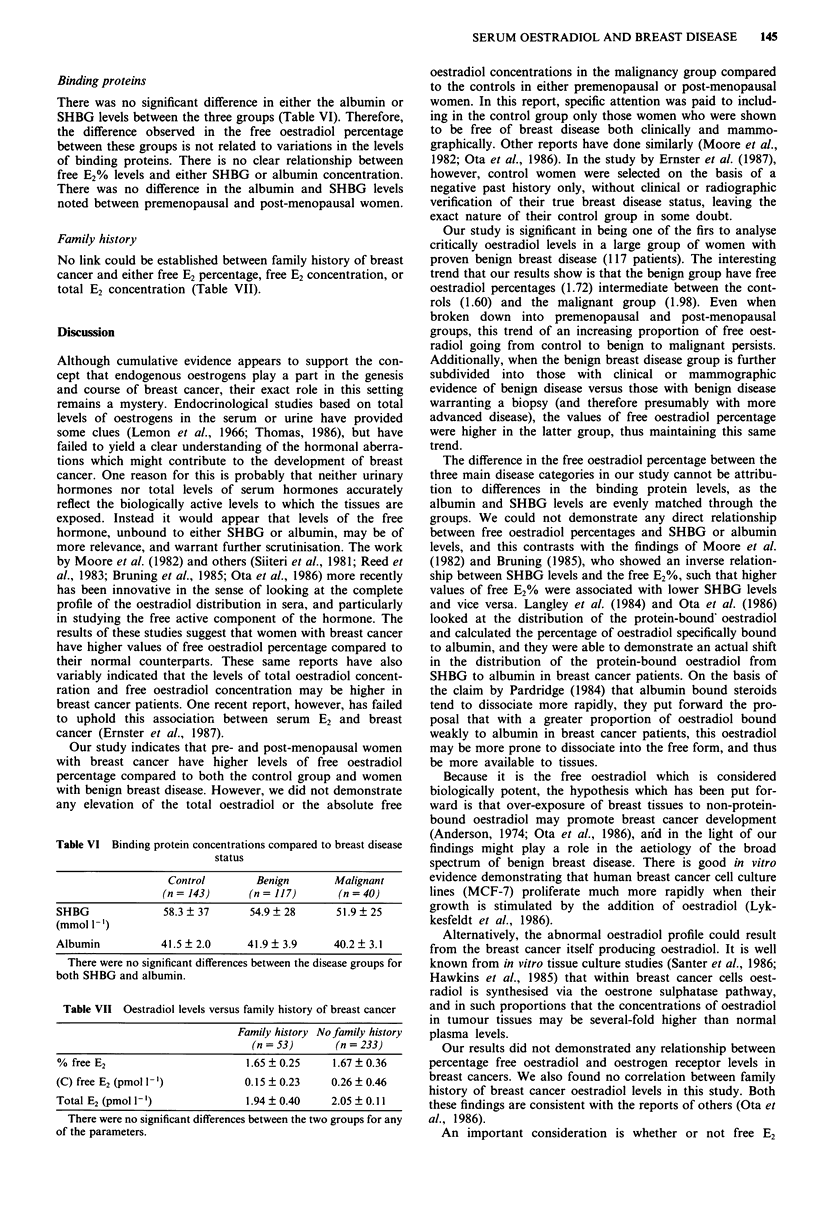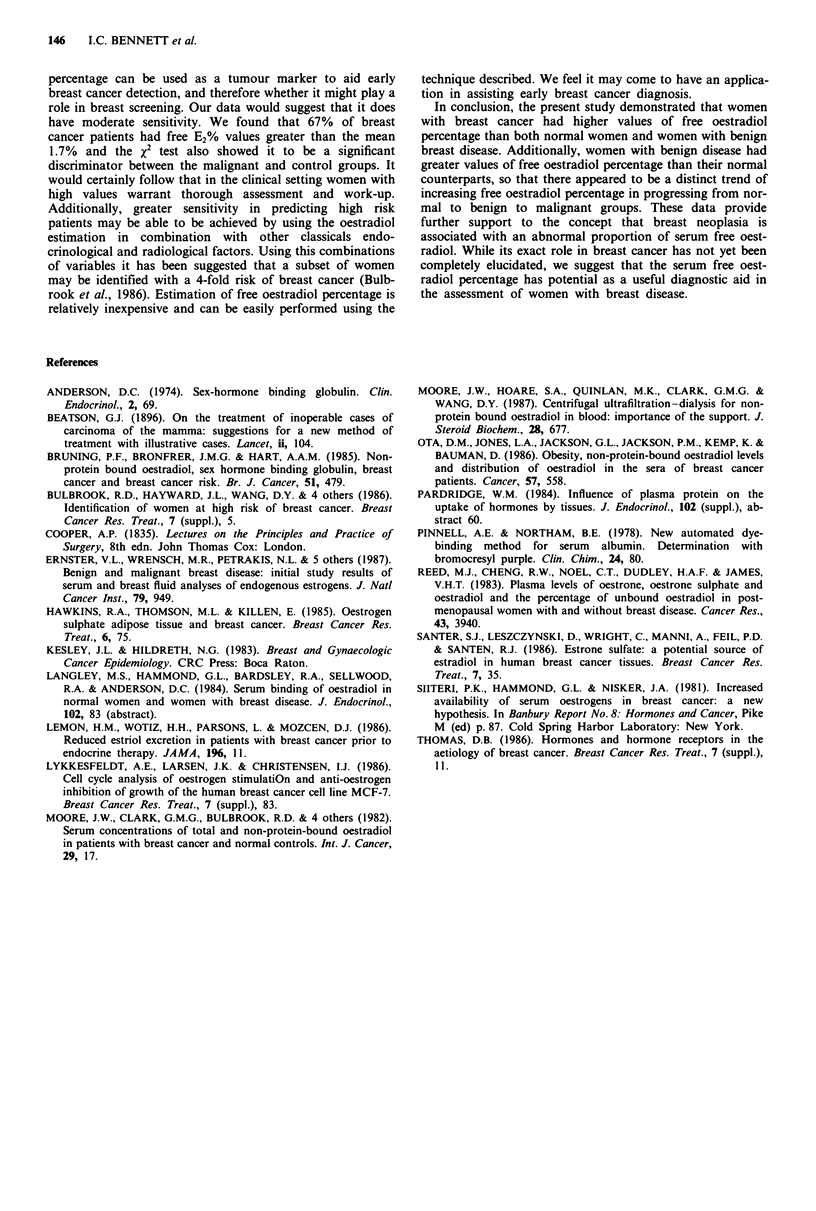# Serum oestradiol in women with and without breast disease.

**DOI:** 10.1038/bjc.1990.29

**Published:** 1990-01

**Authors:** I. C. Bennett, J. F. McCaffrey, E. McCaffrey, B. Wyatt

**Affiliations:** Department of Surgery, University of Queensland, Royal Brisbane Hospital, Herston, Australia.

## Abstract

It has been suggested that the percentage of non-protein-bound or free oestradiol (E2) is abnormally high in patients with breast cancer. In this study, the serum oestradiol profiles of a large group of women were analysed to determine whether a significant correlation could be found between serum oestradiol and various breast diseases. In addition oestradiol levels were measured in relation to sex hormone binding globulin (SHBG), albumin levels, oestrogen receptor status and family history of breast cancer. Serum samples were taken from a total of 300 women who had either no breast disease, benign breast disease or breast cancer. The percentage of free oestradiol was found to be highest in women with breast cancer, lowest in the control group and intermediate for the women with benign breast disease. These differences were most marked in post-menopausal women. The absolute values for total and free oestradiol were not statistically different in the three groups studied. There did not appear to be a correlation between oestrogen receptor (ER) concentration in breast cancer tissue and free E2 percentage levels. Women who had a family history of breast cancer did not appear to have higher percentage levels of free E2 than those with no such history. The presence of elevated proportions of free oestradiol in the serum of women with breast cancer may be significant in regard to understanding the aetiology of breast neoplasia. There also may be important implications for the use of this measurement in the earlier diagnosis and detection of breast cancer.


Br. J. Cancer (1990), 61, 142-146 © Macmillan Press Ltd., 1990

### Serum oestradiol in women with and without breast disease

I.C. Bennett, J.F. McCaffrey, E. McCaffrey & B. Wyatt

*Department of Surgery, University of Queensland, Royal Brisbane Hospital, Herston, Queensland, Australia.*

**Summary** It has been suggested that the percentage of non-protein-bound or free oestradiol ($E_2$) is abnormally high in patients with breast cancer. In this study, the serum oestradiol profiles of a large group of women were analysed to determine whether a significant correlation could be found between serum oestradiol and various breast diseases. In addition oestradiol levels were measured in relation to sex hormone binding globulin (SHBG), albumin levels, oestrogen receptor status and family history of breast cancer. Serum samples were taken from a total of 300 women who had either no breast disease, benign breast disease or breast cancer. The percentage of free oestradiol was found to be highest in women with breast cancer, lowest in the control group and intermediate for the women with benign breast disease. These differences were most marked in post-menopausal women. The absolute values for total and free oestradiol were not statistically different in the three groups studied. There did not appear to be a correlation between oestrogen receptor (ER) concentration in breast cancer tissue and free $E_2$ percentage levels. Women who had a family history of breast cancer did not appear to have higher percentage levels of free $E_2$ than those with no such history. The presence of elevated proportions of free oestradiol in the serum of women with breast cancer may be significant in regard to understanding the aetiology of breast neoplasia. There also may be important implications for the use of this measurement in the earlier diagnosis and detection of breast cancer.

Endogenous hormones, in particular oestrogens, have long been recognised as having a fundamental role in the development and progress of breast cancer. As early as 1835 Sir Astley Cooper made the observation that nulliparous women were more likely to develop breast cancer, and in 1896 George Beatson in Glasgow demonstrated that oophorectomy could induce remission of advanced breast cancer in premenopausal patients.

In more recent times a role for oestrogens in the aetiology of breast cancer has been suggested by numerous laboratory, epidemiological and clinical studies (Kelsey & Hildreth, 1983). Animal studies have demonstrated that oestrogens can induce and promote mammary tumours in rodent. Epidemiological evidence concerning this role includes the rarity of the disease in males, the increased risk associated with nulliparity, a late age at first live birth, an early age of menarche and a late age at menopause, and a decreased risk following oophorectomy. Endocrine ablation therapy including oophorectomy, adrenalectomy, hypophysectomy and administration of anti-oestrogens may lead to regression of breast cancer. However, the underlying biological relationship between breast cancers and oestrogens still remains poorly understood.

Lately attention has focused on one of the forms of oestrogen, oestradiol, which exists in the serum in both a bound form (bound to either SHBG or albumin) and in an unbound from which is the biologically active component. Studies have recently suggested that serum oestradiol levels, and particularly the percentage of free oestradiol, may be higher in breast cancer patients than in matched controls (Siiteri *et al.*, 1981; Moore *et al.*, 1982; Reed *et al.*, 1983; Ota *et al.*, 1986). This work suggested that the greater availability of free oestradiol may provide a stimulus to breast cancer development. The original work by Moore *et al.* (1982) has received some support from other centres. The explanation for the elevation in the free oestradiol fraction remains unclear.

We have examined the serum oestradiol profiles of a group of 300 women in order to assess the relationship between oestradiol levels and breast disease. In addition to comparing both a control group and a group of women with breast cancer, we have also studied a subset of 117 women with proven benign breast disease. This is one of the largest study groups so far reported of women with proven benign breast disease in whom the serum oestradiol profiles has been analysed.

Total serum oestradiol, free oestradiol concentration and the free oestradiol percentage have been measured in all patients. The relationship between free oestradiol percentage and breast disease, SHBG and albumin levels, oestrogen receptor status and family history of breast cancer has been evaluated.

Correspondence: J.F. McCaffrey.
Received 11 April 1989; and in revised form 11 September 1989.

### Subjects and methods

### *Subjects*

Blood was collected by venepuncture from a total of 300 women who attended either a breast screening clinic at the Royal Women's Hospital or were to have an operation at the Royal Brisbane Hospital. On the basis of clinical findings, mammography and histology where relevant, three categories were established, a control group, patients with benign breast disease and patients with breast cancer.

The control group consisted of asymptomatic, well women attending the Royal Women's Hospital breast screening clinic who were found to have no breast disease at all, having a normal clinical examination and normal mammogram.

The benign breast disease group was made up of: (i) women with clinical or mammographic evidence of benign disease; or (ii) women who had a biopsy with the histological diagnosis of benign disease.

Patients with breast cancer were those having operative procedures undertaken with positive histological evidence of the malignancy. In some cases this was confined to a trucut biopsy in patients being treated by methods other than surgical. The oestrogen receptor (ER) values for all the tumours was also recorded.

Information was collected from the patients concerning menopausal status, family history of breast cancer, thyroid function, and medication regimens (including the use of oral contraceptives and hormone replacement therapy). Height and weight were recorded, and a Quetelét index ($kg\,m^{-2}$) was calculated for each patient. Each cancer patient was matched for weight and age with a control patient and a patient with benign breast disease.

In premenopausal patients, blood samples were not taken at any predetermined time in the menstrual cycle as our previous work had shown that the percentage of oestradiol was not significantly altered during the cycle.

*Methods of blood sampling*

Twenty ml of blood were taken from each individual and collected in sterile clotting tubes. Serum was separated by centrifugation and stored at $-20°C$ before analysis. Patients undergoing breast biopsy or surgical treatment for proven breast cancer had samples taken before the operative procedure.

*Analytical methods*

The percentage of free oestradiol was determined using a centrifugal ultrafiltration dialysis technique (Moore *et al.*, 1987). The reagents used in the experimental protocol were D-U-$^{14}$C-glucose (250 mCi mmol$^{-1}$) and 6,7-$^{3}$H-oestradiol (48.2 Ci mmol$^{-1}$), which were obtained from New England Corporation (Boston, MA, USA). Working solutions of the labels were prepared immediately before use.

Undiluted serum (450 µl) was incubated with $3 \times 10^5$ d.p.m. $^3$H-oestradiol-17-β and $12 \times 10^3$ d.p.m. $^{14}$C-glucose for 30 min at 37°C and at room temperature for a further 30 min. Separation of the free and protein-bound ligand was achieved using the AMICON MPS-1 centifree micropartition system. Aliquots (200 µl) of the reaction mixture were transferred to duplicate MPS-1 devices and centrifuged at 2,000 *g* for 30 min at 37°C. The ultrafiltrate (non-protein-bound fraction) was retained and the retentate (protein-bound fraction) obtained on washing of the ultrafiltration membrane with distilled water and recentrifugation. Both the ultrafiltrate and retentate were taken up in a Pico-flor-15 liquid scintillation spectrophotometer adjusted for simultaneous measurement of $^3$H and $^{14}$C. The ratio of $^3$H-oestradiol and $^{14}$C-glucose in the ultrafiltrate and retentate was used to calculate the percentage of non-protein-bound oestradiol (% free $E_2$) using the following formula:

$$\%\text{ free } E_2 = \frac{^3\text{H-E}_2\text{ c.p.m.}}{\text{Ultrafiltrate }^{14}\text{C-glucose c.p.m.}} \div \frac{^3\text{H-E}_2\text{ c.p.m.}}{\text{Retentate }^{14}\text{C-glucose c.p.m.}} \times 100$$

Inter-assay and intra-assay co-efficients of variance using this procedure were 9.8% and 8.3% respectively.

Total oestradiol concentration was measured by conventional radioimmunassay. The free oestradiol concentration was then calculated from the derived values of total oestradiol and free oestradiol.

The two proteins important in binding oestradiol are sex hormone binding globulin (SHBG), because of its high affinity, and albumin, because of the large amount available. The concentrations of these proteins were measured to see whether variations in one or both could account for the differences in free oestradiol percentage. SHBG levels were determined using a radioimmunoassay technique (Anderson, 1974). Serum albumin was measured using bromo-cresol-green dye and automated colour metric analyser (Pinnel *et al.*, 1978).

*Statistics*

Data have been expressed in terms of the mean ± standard deviation. The significance of differences between mean values was determined using the Student's *t* test. A probability of $P<0.05$ was regarded as statistically significant. Correlation between assays was tested using bio-regression analysis using the method of least squares.

### Results

*Patient characteristics*

A total of 300 women were included in the study: 143 women in the control group, 117 women with benign breast disease and 40 women with breast cancer. Of these, 110 women were premenopausal and 190 were post-menopausal. In the group with cancer, eight were premenopausal and 32 were post-menopausal.

Age, height, weight and Queteléť index for each group are shown in Table I. Women with malignancy were of a slightly older age group. They also had a higher mean weight, but when height was also taken into account and the Queteléť index estimated, the three groups were evenly matched.

The number of patients on the contraceptive pill or on hormonal replacement therapy was low and numbers were even through the three groups. Only a few patients had a past history of thyroid disease and all had adequate hormone replacement.

*Percentage free oestradiol*

*Total population* In the study population the mean free oestradiol percentage was 1.70 with a standard deviation of 0.4. Within the breast cancer group, the mean free oestradiol percentage was 1.98 (± 0.5) which was significantly higher than the control group 1.60 (± 0.4) ($P<0.001$) and women with benign breast disease 1.72 (± 0.4) ($P<0.01$) (see Table II). These same trends persisted when the control and benign patients were weight and age matched with cancer patients (Table III). An important finding is that 67% of breast cancer patients had a percentage free $E_2$ level greater than the mean value of 1.70. The frequency of patients with malignant disease having percentage free $E_2$ values greater than that for normal women ($\chi^2 = 13.54$, 5 d.f., $0.05>P>0.01$).

Further subdivision of the benign breast disease group was done into those with clinical and/or mammographic evidence of benign disease and those with benign disease requiring surgical biopsy because of a concern about the possibility of malignancy. It was found that the biopsy group had a higher level of free oestradiol percentage than the former (Table IV). Thus there is an incremental trend present in progressing from the control group to the biopsy benign group to the carcinoma group.

*Menopausal status* When menopausal status was taken into account (Table V), the same trend of increasing free oestradiol percentage from control to benign malignant is observed. The difference was significant in the post-menopausal group ($P<0.001$), and was clearly present but not as statistically strong ($P=0.056$) in the premenopausal cancer patients. The mean free oestradiol percentage in premenopausal women was 1.73 (± 0.4), not significantly different from that of post-menopausal women, 1.68 (± 0.4) ($P<0.36$).

*Weight* There was no significant correlation between free $E_2$% and weight ($r$ value = − 0.015). Although patients in the malignancy group tended to have higher weights, the higher values of free $E_2$% in this group were not related to this.

*ER status* There was no significant relationship between the oestrogen receptor value (fmol mg$^{-1}$ protein) and free oestradiol percentage ($r = -0.142$).

*Concentrations of total and free oestradiol*

There was no significant difference in the absolute values of free $E_2$ or total $E_2$ in patients with breast cancer when compared with either the control group or women with benign breast disease (Tables II and III).

Even when the groups are subdivided into premenopausal and post-menopausal, no clear trend could be noted between the control, benign and malignant groups for either free oestradiol or total oestradiol (Table V).

Premenopausal women have higher values for both mean free oestradiol concentration and total oestradiol concentration than do post-menopausal women ($P<0.0001$ both groups).

**Table I** Characteristics of total population grouped according to disease status

| *Parameter* | *Control* | *Benign* | *Malignant* |
|---|---|---|---|
| No. of patients | 143 | 117 | 40 |
| Age (years, mean ± s.d.) | 62.77 ± 11.18 | 61.73 ± 10.48 | 68.16 ± 11.95 |
| Weight (kg, mean ± s.d.) | 53.53 ± 11.65 | 51.08 ± 10.61 | 62.00 ± 13.71 |
| Mean QI | 23.84 | 23.48 | 22.45 |
| (kg $m^{-2}$) (range) | (17.53–40.79) | (19.66–28.58) | (18.56–24.56) |
| Contraceptive pill | 5 | 3 | 2 |
| HR therapy | 7 | 6 | 1 |
| Thyroid disease | 11 | 5 | 2 |

**Table II** Total, free and percentage free oestradiol values versus breast disease status for total population

| | | *Breast disease status* | |
|---|---|---|---|
| *Parameter* | *Control (n = 143)* | *Benign (n = 117)* | *Malignant (n = 40)* |
| Total $E_2$ (pmol $l^{-1}$) | 181 ± 316 | 246 ± 327 | 117 ± 212 |
| $\log_{10}$ Total $E_2$ | 1.96 ± 0.4 | 2.03 ± 0.5 | 1.85 ± 0.4 |
| (C) free $E_2$ (pmol $l^{-1}$) | 3.2 ± 6.5 | 4.5 ± 6.3 | 2.5 ± 4.4 |
| $\log_{10}$ (C) free $E_2$ | 0.08 ± 0.3 | 0.25 ± 0.5 | 0.16 ± 0.4 |
| % free $E_2$ | 1.60 ± 0.4 | 1.72 ± 0.4 | 1.98 ± 0.5 |

The % free $E_2$ recorded for women with malignant disease is significantly higher than that recorded for normal controls ($P<0.001$) and benign disease ($P<0.01$).

**Table III** Oestradiol values versus breast disease status with control and benign disease patients matched weight and age for each cancer patient

| | | *Breast disease status* | |
|---|---|---|---|
| *Parameter* | *Control (n = 40)* | *Benign (n = 40)* | *Malignant (n = 40)* |
| $\text{Log}_{10}$ total $E_2$ | 1.74 ± 0.3 | 1.88 ± 0.4 | 1.85 ± 0.4 |
| $\text{Log}_{10}$ (C) free $E_2$ | 0.07 ± 0.3 | 0.07 ± 0.4 | 0.16 ± 0.4[a] |
| % free $E_2$ | 1.58 ± 0.3 | 1.60 ± 0.4 | 1.98 ± 0.5[b,c] |

Serum oestradiol levels for normal controls and patients with benign breast disease matched for weight and age with each cancer patient. The free oestradiol and total oestradiol levels are expressed as $\log_{10}$ values. Significance levels where appropriate are indicated on the table by the following symbols. [a]Malignant disease significantly greater than normal controls $P<0.01$. [b]Malignant disease significantly greater than normal controls $P<0.001$. [c]Malignant disease significantly greater than benign disease $P<0.001$.

**Table IV** Percentage free $E_2$ versus breast disease status

| | *Control (n = 143)* | *Clinically benign (n = 96)* | *Biopsy benign (n = 21)* | *Malignant (n = 40)* |
|---|---|---|---|---|
| Free $E_2$% | 1.60 ± 0.4 | 1.70 ± 0.4 | 1.82 ± 0.4 | 1.98 ± 0.5 |

The % free oestradiol levels for women classified according to breast disease status. The value recorded for women with malignant breast disease was significantly higher than that recorded for normal controls and patients with clinically benign disease ($P<0.001$), but not for biopsy benign patients. Similarly, biopsy benign patients had significantly higher % free oestradiol levels than normal controls ($P<0.05$) but not patients with clinically benign disease.

**Table V** Oestradiol levels versus breast disease status in premenopausal and post-menopausal population

| | | *Breast disease status* | |
|---|---|---|---|
| *Parameter* | *Control (n = 143)* | *Benign (n = 117)* | *Malignant (n = 40)* |
| *Premenopausal* | | | |
| Total $E_2$ (pmol $l^{-1}$) | 395 ± 455 | 338 ± 356 | 329 ± 371 |
| $\log_{10}$ total $E_2$ | 2.3 ± 0.5 | 2.4 ± 0.4 | 2.3 ± 0.4 |
| (C) free $E_2$ | 7.33 ± 9.5 | 6.08 ± 7.1 | 6.89 ± 7.01 |
| % free $E_2$ | 1.65 ± 0.3 | 1.75 ± 0.4 | 2.01 ± 0.5 ($P = 0.056$) |
| *Post-menopausal* | | | |
| Total $E_2$ (pmol $l^{-1}$) | 77 ± 110 | 160 ± 257 | 64 ± 53 |
| $\log_{10}$ total $E_2$ | 1.8 ± 0.7 | 1.8 ± 0.4 | 1.7 ± 0.2 |
| (C) free $E_2$ (pmol $l^{-1}$) | 0.02 ± 0.4 | 0.07 ± 0.4 | 1.20 ± 1.1 |
| $\log_{10}$ (C) free $E_2$ | 0.02 ± 0.4 | 0.07 ± 0.4 | 0.01 ± 0.3 |
| % free $E_2$ | 1.58 ± 0.4 | 1.69 ± 0.4 | 1.97 ± 0.5 ($P<0.001$) |

The % free $E_2$ levels in post-menopausal women with malignant disease were significantly higher than those for normal controls and patients with benign disease ($P<0.001$). There were no other differences of statistical significance.

*Binding proteins*

There was no significant difference in either the albumin or SHBG levels between the three groups (Table VI). Therefore, the difference observed in the free oestradiol percentage between these groups is not related to variations in the levels of binding proteins. There is no clear relationship between free $E_2$% levels and either SHBG or albumin concentration. There was no difference in the albumin and SHBG levels noted between premenopausal and post-menopausal women.

*Family history*

No link could be established between family history of breast cancer and either free $E_2$ percentage, free $E_2$ concentration, or total $E_2$ concentration (Table VII).

### Discussion

Although cumulative evidence appears to support the concept that endogenous oestrogens play a part in the genesis and course of breast cancer, their exact role in this setting remains a mystery. Endocrinological studies based on total levels of oestrogens in the serum or urine have provided some clues (Lemon *et al.*, 1966; Thomas, 1986), but have failed to yield a clear understanding of the hormonal aberrations which might contribute to the development of breast cancer. One reason for this is probably that neither urinary hormones nor total levels of serum hormones accurately reflect the biologically active levels to which the tissues are exposed. Instead it would appear that levels of the free hormone, unbound to either SHBG or albumin, may be of more relevance, and warrant further scrutinisation. The work by Moore *et al.* (1982) and others (Siiteri *et al.*, 1981; Reed *et al.*, 1983; Bruning *et al.*, 1985; Ota *et al.*, 1986) more recently has been innovative in the sense of looking at the complete profile of the oestradiol distribution in sera, and particularly in studying the free active component of the hormone. The results of these studies suggest that women with breast cancer have higher values of free oestradiol percentage compared to their normal counterparts. These same reports have also variably indicated that the levels of total oestradiol concentration and free oestradiol concentration may be higher in breast cancer patients. One recent report, however, has failed to uphold this association between serum $E_2$ and breast cancer (Ernster *et al.*, 1987).

Our study indicates that pre- and post-menopausal women with breast cancer have higher levels of free oestradiol percentage compared to both the control group and women with benign breast disease. However, we did not demonstrate any elevation of the total oestradiol or the absolute free oestradiol concentrations in the malignancy group compared to the controls in either premenopausal or post-menopausal women. In this report, specific attention was paid to including in the control group only those women who were shown to be free of breast disease both clinically and mammographically. Other reports have done similarly (Moore *et al.*, 1982; Ota *et al.*, 1986). In the study by Ernster *et al.* (1987), however, control women were selected on the basis of a negative past history only, without clinical or radiographic verification of their true breast disease status, leaving the exact nature of their control group in some doubt.

Our study is significant in being one of the firs to analyse critically oestradiol levels in a large group of women with proven benign breast disease (117 patients). The interesting trend that our results show is that the benign group have free oestradiol percentages (1.72) intermediate between the controls (1.60) and the malignant group (1.98). Even when broken down into premenopausal and post-menopausal groups, this trend of an increasing proportion of free oestradiol going from control to benign to malignant persists. Additionally, when the benign breast disease group is further subdivided into those with clinical or mammographic evidence of benign disease versus those with benign disease warranting a biopsy (and therefore presumably with more advanced disease), the values of free oestradiol percentage were higher in the latter group, thus maintaining this same trend.

The difference in the free oestradiol percentage between the three main disease categories in our study cannot be attribution to differences in the binding protein levels, as the albumin and SHBG levels are evenly matched through the groups. We could not demonstrate any direct relationship between free oestradiol percentages and SHBG or albumin levels, and this contrasts with the findings of Moore *et al.* (1982) and Bruning (1985), who showed an inverse relationship between SHBG levels and the free $E_2$%, such that higher values of free $E_2$% were associated with lower SHBG levels and vice versa. Langley *et al.* (1984) and Ota *et al.* (1986) looked at the distribution of the protein-bound' oestradiol and calculated the percentage of oestradiol specifically bound to albumin, and they were able to demonstrate an actual shift in the distribution of the protein-bound oestradiol from SHBG to albumin in breast cancer patients. On the basis of the claim by Pardridge (1984) that albumin bound steroids tend to dissociate more rapidly, they put forward the proposal that with a greater proportion of oestradiol bound weakly to albumin in breast cancer patients, this oestradiol may be more prone to dissociate into the free form, and thus be more available to tissues.

Because it is the free oestradiol which is considered biologically potent, the hypothesis which has been put forward is that over-exposure of breast tissues to non-protein-bound oestradiol may promote breast cancer development (Anderson, 1974; Ota *et al.*, 1986), and in the light of our findings might play a role in the aetiology of the broad spectrum of benign breast disease. There is good *in vitro* evidence demonstrating that human breast cancer cell culture lines (MCF-7) proliferate much more rapidly when their growth is stimulated by the addition of oestradiol (Lykkesfeldt *et al.*, 1986).

Alternatively, the abnormal oestradiol profile could result from the breast cancer itself producing oestradiol. It is well known from *in vitro* tissue culture studies (Santer *et al.*, 1986; Hawkins *et al.*, 1985) that within breast cancer cells oestradiol is synthesised via the oestrone sulphatase pathway, and in such proportions that the concentrations of oestradiol in tumour tissues may be several-fold higher than normal plasma levels.

Our results did not demonstrated any relationship between percentage free oestradiol and oestrogen receptor levels in breast cancers. We also found no correlation between family history of breast cancer oestradiol levels in this study. Both these findings are consistent with the reports of others (Ota *et al.*, 1986).

An important consideration is whether or not free $E_2$

**Table VI** Binding protein concentrations compared to breast disease status

| | *Control* ($n = 143$) | *Benign* ($n = 117$) | *Malignant* ($n = 40$) |
|---|---|---|---|
| SHBG (mmol $l^{-1}$) | 58.3 ± 37 | 54.9 ± 28 | 51.9 ± 25 |
| Albumin | 41.5 ± 2.0 | 41.9 ± 3.9 | 40.2 ± 3.1 |

There were no significant differences between the disease groups for both SHBG and albumin.

**Table VII** Oestradiol levels versus family history of breast cancer

| | *Family history* ($n = 53$) | *No family history* ($n = 233$) |
|---|---|---|
| % free $E_2$ | 1.65 ± 0.25 | 1.67 ± 0.36 |
| (C) free $E_2$ (pmol $l^{-1}$) | 0.15 ± 0.23 | 0.26 ± 0.46 |
| Total $E_2$ (pmol $l^{-1}$) | 1.94 ± 0.40 | 2.05 ± 0.11 |

There were no significant differences between the two groups for any of the parameters.

percentage can be used as a tumour marker to aid early breast cancer detection, and therefore whether it might play a role in breast screening. Our data would suggest that it does have moderate sensitivity. We found that 67% of breast cancer patients had free $E_2$% values greater than the mean 1.7% and the $\chi^2$ test also showed it to be a significant discriminator between the malignant and control groups. It would certainly follow that in the clinical setting women with high values warrant thorough assessment and work-up. Additionally, greater sensitivity in predicting high risk patients may be able to be achieved by using the oestradiol estimation in combination with other classicals endocrinological and radiological factors. Using this combinations of variables it has been suggested that a subset of women may be identified with a 4-fold risk of breast cancer (Bulbrook *et al.*, 1986). Estimation of free oestradiol percentage is relatively inexpensive and can be easily performed using the technique described. We feel it may come to have an application in assisting early breast cancer diagnosis.

In conclusion, the present study demonstrated that women with breast cancer had higher values of free oestradiol percentage than both normal women and women with benign breast disease. Additionally, women with benign disease had greater values of free oestradiol percentage than their normal counterparts, so that there appeared to be a distinct trend of increasing free oestradiol percentage in progressing from normal to benign to malignant groups. These data provide further support to the concept that breast neoplasia is associated with an abnormal proportion of serum free oestradiol. While its exact role in breast cancer has not yet been completely elucidated, we suggest that the serum free oestradiol percentage has potential as a useful diagnostic aid in the assessment of women with breast disease.

### References

ANDERSON, D.C. (1974). Sex-hormone binding globulin. *Clin. Endocrinol.*, **2**, 69.

BEATSON, G.J. (1896). On the treatment of inoperable cases of carcinoma of the mamma: suggestions for a new method of treatment with illustrative cases. *Lancet*, **ii**, 104.

BRUNING, P.F., BRONFRER, J.M.G. & HART, A.A.M. (1985). Non-protein bound oestradiol, sex hormone binding globulin, breast cancer and breast cancer risk. *Br. J. Cancer*, **51**, 479.

BULBROOK, R.D., HAYWARD, J.L., WANG, D.Y. & 4 others (1986). Identification of women at high risk of breast cancer. *Breast Cancer Res. Treat.*, **7** (suppl.), 5.

COOPER, A.P. (1835). *Lectures on the Principles and Practice of Surgery*, 8th edn. John Thomas Cox: London.

ERNSTER, V.L., WRENSCH, M.R., PETRAKIS, N.L. & 5 others (1987). Benign and malignant breast disease: initial study results of serum and breast fluid analyses of endogenous estrogens. *J. Natl Cancer Inst.*, **79**, 949.

HAWKINS, R.A., THOMSON, M.L. & KILLEN, E. (1985). Oestrogen sulphate adipose tissue and breast cancer. *Breast Cancer Res. Treat.*, **6**, 75.

KESLEY, J.L. & HILDRETH, N.G. (1983). *Breast and Gynaecologic Cancer Epidemiology*. CRC Press: Boca Raton.

LANGLEY, M.S., HAMMOND, G.L., BARDSLEY, R.A., SELLWOOD, R.A. & ANDERSON, D.C. (1984). Serum binding of oestradiol in normal women and women with breast disease. *J. Endocrinol.*, **102**, 83 (abstract).

LEMON, H.M., WOTIZ, H.H., PARSONS, L. & MOZCEN, D.J. (1986). Reduced estriol excretion in patients with breast cancer prior to endocrine therapy. *JAMA*, **196**, 11.

LYKKESFELDT, A.E., LARSEN, J.K. & CHRISTENSEN, I.J. (1986). Cell cycle analysis of oestrogen stimulatiOn and anti-oestrogen inhibition of growth of the human breast cancer cell line MCF-7. *Breast Cancer Res. Treat.*, **7** (suppl.), 83.

MOORE, J.W., CLARK, G.M.G., BULBROOK, R.D. & 4 others (1982). Serum concentrations of total and non-protein-bound oestradiol in patients with breast cancer and normal controls. *Int. J. Cancer*, **29**, 17.

MOORE, J.W., HOARE, S.A., QUINLAN, M.K., CLARK, G.M.G. & WANG, D.Y. (1987). Centrifugal ultrafiltration–dialysis for non-protein bound oestradiol in blood: importance of the support. *J. Steroid Biochem.*, **28**, 677.

OTA, D.M., JONES, L.A., JACKSON, G.L., JACKSON, P.M., KEMP, K. & BAUMAN, D. (1986). Obesity, non-protein-bound oestradiol levels and distribution of oestradiol in the sera of breast cancer patients. *Cancer*, **57**, 558.

PARDRIDGE, W.M. (1984). Influence of plasma protein on the uptake of hormones by tissues. *J. Endocrinol.*, **102** (suppl.), abstract 60.

PINNELL, A.E. & NORTHAM, B.E. (1978). New automated dye-binding method for serum albumin. Determination with bromocresyl purple. *Clin. Chim.*, **24**, 80.

REED, M.J., CHENG, R.W., NOEL, C.T., DUDLEY, H.A.F. & JAMES, V.H.T. (1983). Plasma levels of oestrone, oestrone sulphate and oestradiol and the percentage of unbound oestradiol in post-menopausal women with and without breast disease. *Cancer Res.*, **43**, 3940.

SANTER, S.J., LESZCZYNSKI, D., WRIGHT, C., MANNI, A., FEIL, P.D. & SANTEN, R.J. (1986). Estrone sulfate: a potential source of estradiol in human breast cancer tissues. *Breast Cancer Res. Treat.*, **7**, 35.

SIITERI, P.K., HAMMOND, G.L. & NISKER, J.A. (1981). Increased availability of serum oestrogens in breast cancer: a new hypothesis. In *Banbury Report No. 8: Hormones and Cancer*, Pike M (ed) p. 87. Cold Spring Harbor Laboratory: New York.

THOMAS, D.B. (1986). Hormones and hormone receptors in the aetiology of breast cancer. *Breast Cancer Res. Treat.*, **7** (suppl.), 11.