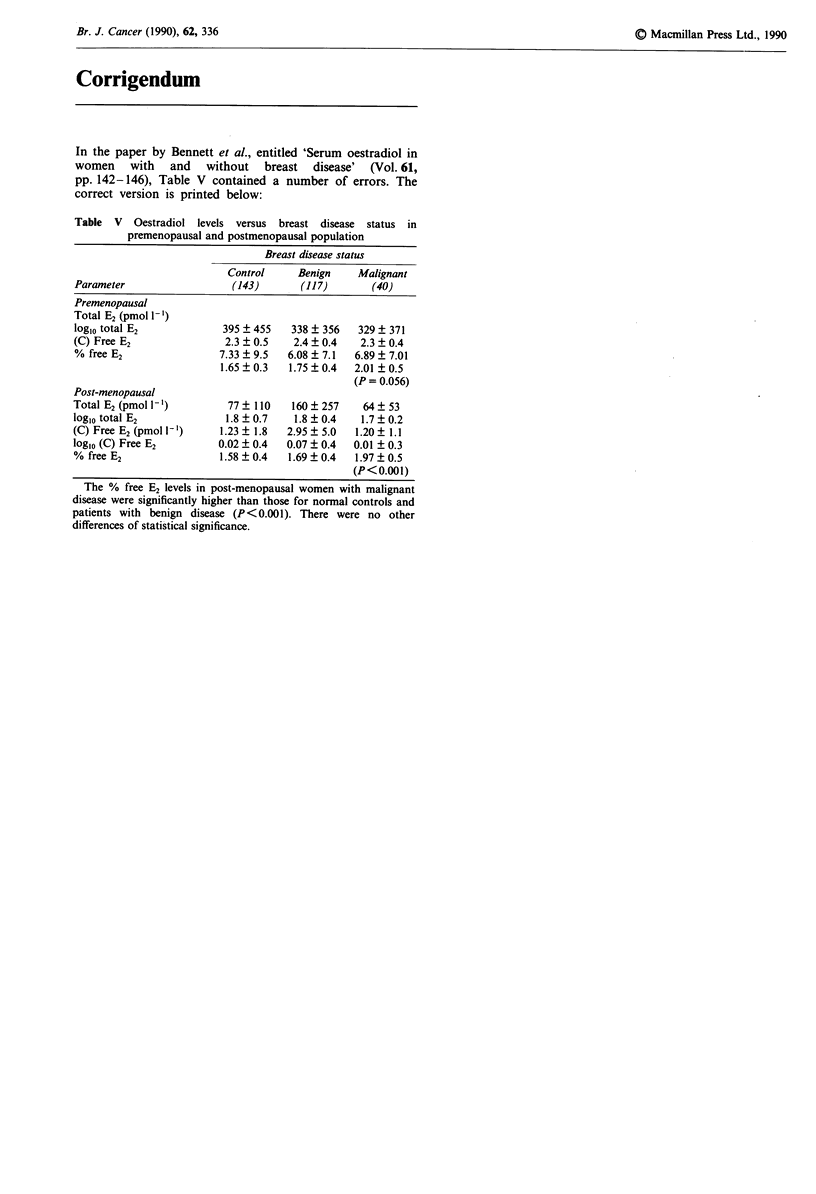# Corrigendum

**Published:** 1990-08

**Authors:** 


*Br. J. Cancer* (1990), **62**, 336 © Macmillan Press Ltd., 1990

### Corrigendum

In the paper by Bennett *et al.*, entitled 'Serum oestradiol in women with and without breast disease' (Vol. **61**, pp. 142–146), Table V contained a number of errors. The correct version is printed below:

**Table V** Oestradiol levels versus breast disease status in premenopausal and postmenopausal population

| | *Breast disease status* | | |
|---|---|---|---|
| *Parameter* | *Control (143)* | *Benign (117)* | *Malignant (40)* |
| *Premenopausal* | | | |
| Total $E_2$ (pmol $l^{-1}$) | | | |
| $\log_{10}$ total $E_2$ | $395 \pm 455$ | $338 \pm 356$ | $329 \pm 371$ |
| (C) Free $E_2$ | $2.3 \pm 0.5$ | $2.4 \pm 0.4$ | $2.3 \pm 0.4$ |
| % free $E_2$ | $7.33 \pm 9.5$ | $6.08 \pm 7.1$ | $6.89 \pm 7.01$ |
| | $1.65 \pm 0.3$ | $1.75 \pm 0.4$ | $2.01 \pm 0.5$ |
| | | | ($P = 0.056$) |
| *Post-menopausal* | | | |
| Total $E_2$ (pmol $l^{-1}$) | $77 \pm 110$ | $160 \pm 257$ | $64 \pm 53$ |
| $\log_{10}$ total $E_2$ | $1.8 \pm 0.7$ | $1.8 \pm 0.4$ | $1.7 \pm 0.2$ |
| (C) Free $E_2$ (pmol $l^{-1}$) | $1.23 \pm 1.8$ | $2.95 \pm 5.0$ | $1.20 \pm 1.1$ |
| $\log_{10}$ (C) Free $E_2$ | $0.02 \pm 0.4$ | $0.07 \pm 0.4$ | $0.01 \pm 0.3$ |
| % free $E_2$ | $1.58 \pm 0.4$ | $1.69 \pm 0.4$ | $1.97 \pm 0.5$ |
| | | | ($P < 0.001$) |

The % free $E_2$ levels in post-menopausal women with malignant disease were significantly higher than those for normal controls and patients with benign disease ($P < 0.001$). There were no other differences of statistical significance.